# Angiogenesis in Pituitary Adenomas: Human Studies and New Mutant Mouse Models

**DOI:** 10.1155/2014/608497

**Published:** 2014-11-18

**Authors:** Carolina Cristina, Guillermina María Luque, Gianina Demarchi, Felicitas Lopez Vicchi, Lautaro Zubeldia-Brenner, Maria Ines Perez Millan, Sofia Perrone, Ana Maria Ornstein, Isabel M. Lacau-Mengido, Silvia Inés Berner, Damasia Becu-Villalobos

**Affiliations:** ^1^Instituto de Biología y Medicina Experimental, CONICET, Vuelta de Obligado 2490, 1428 Buenos Aires, Argentina; ^2^CITNOBA (CONICET-UNNOBA), Universidad Nacional del Noroeste de la Provincia de Buenos Aires, Monteagudo 2772, Pergamino, 2700 Buenos Aires, Argentina; ^3^Servicio de Neurocirugía, Clínica Santa Isabel, Avenida Directorio 2037, C1406GZJ Buenos Aires, Argentina; ^4^Servicio de Neurocirugía, Hospital Santa Lucía, Avenida San Juan 2021, C1232AAC Buenos Aires, Argentina

## Abstract

The role of angiogenesis in pituitary tumor development has been questioned, as pituitary tumors have been usually found to be less vascularized than the normal pituitary tissue. Nevertheless, a significantly higher degree of vasculature has been shown in invasive or macropituitary prolactinomas when compared to noninvasive and microprolactinomas. Many growth factors and their receptors are involved in pituitary tumor development. For example, VEGF, FGF-2, FGFR1, and PTTG, which give a particular vascular phenotype, are modified in human and experimental pituitary adenomas of different histotypes. In particular, vascular endothelial growth factor, VEGF, the central mediator of angiogenesis in endocrine glands, was encountered in experimental and human pituitary tumors at different levels of expression and, in particular, was higher in dopamine agonist resistant prolactinomas. Furthermore, several anti-VEGF techniques lowered tumor burden in human and experimental pituitary adenomas. Therefore, even though the role of angiogenesis in pituitary adenomas is contentious, VEGF, making permeable pituitary endothelia, might contribute to adequate temporal vascular supply and mechanisms other than endothelial cell proliferation. The study of angiogenic factor expression in aggressive prolactinomas with resistance to dopamine agonists will yield important data in the search of therapeutical alternatives.

## 1. Introduction

The formation of new blood vessels within neoplasms, termed angiogenesis, provides the tumor tissues with oxygen and basic energetic compounds. An increase in tumor size necessarily requires a corresponding increase in vascularization that is assured by means of this complex dynamic process. In most human tumors, including breast, bladder, and stomach, angiogenesis has been shown to be correlated with tumor behavior [1]. On the other hand, pituitary tumors have been reported to be less vascularized than the normal pituitary tissue [2–4], and differences in the angiogenic pattern of pituitary adenomas have yielded highly controversial results concerning hormonal phenotypes, size, or invasion. In most studies, immunohistochemistry evaluation of different markers of microvascular density (MVD) such as cluster differentiation molecules (CD31 and CD34), Factor VIII (factor eight-related antigen), and Ulex europaeus agglutinin I has been used. Nevertheless, the appraisal of MVD by immunohistochemistry has a number of substantial limitations, which are mainly due to the complex biology of tumor vasculature, and the irregular geometry of the vascular system [5].

Nevertheless, accumulating evidence points to increased angiogenesis in pituitary adenomas. For example, it has been described that macroprolactinomas are significantly more vascular than microprolactinomas [3], and Turner et al. demonstrated a significantly higher degree of vasculature of invasive pituitary prolactinomas [6]. Inhibitors of angiogenesis were effective in the suppression of growth of experimental prolactinomas [7] and, besides, in angiographic studies, the presence of additional arteries (which were not part of the portal system) was found in 66% of patients with pituitary adenomas [8].

Even so, the role of angiogenesis in pituitary tumor development has been questioned, as the normal pituitary is a highly vascularized gland.

In this review we summarize data on angiogenesis in human pituitary adenomas, as well as in two mouse models of dopamine agonist resistant prolactinomas: the dopamine D2 receptor (D2R) knockout mouse (*Drd2* ^−/−^) [9] and the lactotrope specific D2R knockout mouse (lacD2RKO) generated by Cre* LoxP* technology [10].

## 2. Angiogenic Factors in Human Pituitary Adenomas

### 2.1. Vascular Endothelial Growth Factor

Vascular endothelial growth factor-A (VEGF-A or VEGF) is a central regulator of angiogenesis. It is the founding member of a family of closely related cytokines that exert critical functions in vasculogenesis and in both pathologic and physiologic angiogenesis and lymphangiogenesis. The VEGF-A gene is located on the short arm of chromosome 6 and is differentially spliced to yield several different isoforms, the three most prominent of which encode polypeptides of 189, 165, and 121 amino acids in human cells. The protein has a hydrophobic leader sequence and typical of secreted proteins. It was discovered in the late 1970s as a tumor-secreted protein that potently increased microvascular permeability to plasma proteins. It is essential for normal developmental vasculogenesis and angiogenesis, as both null (*VEGF-A* ^−/−^) and heterozygote (*VEGF-A* ^+/−^) animals are embryonic lethals. It increases vascular permeability to plasma and plasma proteins, a characteristic trait of the tumor microvasculature and a critical early step in tumor stroma generation. It is a selective mitogen for vascular endothelium because its major tyrosine kinase receptors are selectively (though not exclusively) expressed on vascular endothelium, and furthermore, it is overexpressed in a variety of human cancer cells (in human vascular tumors, including brain, colon, gastrointestinal tract, ovary, and breast) [1].

Type 2 VEGF receptor (VEGFR2) is the major positive signal transducer for both physiological and pathological angiogenesis induced by VEGF. It is a highly active kinase receptor and triggers a broad spectrum of signaling cascades. The phosphoinositide 3-kinase (PI3K) signal transduction pathway leading to phosphorylation of protein kinase B (AKT) has emerged as one of the main signal routes of VEGFR2 activation [11]. Indeed, many experiments using* in vivo* and* in vitro* systems have demonstrated that activation of PI3K by VEGFR2 promotes endothelial cell survival, proliferation, and angiogenesis, and the overexpression of a dominant-negative form of AKT blocks the survival effect of VEGF [12].

### 2.2. Vascular Endothelial Growth Factor in the Pituitary Gland

The pituitary contains abundant VEGF as well as VEGFR2 [13, 14], and VEGF participates in the formation of the vascular network of a new pituitary tumor [15, 16]. It is also involved in the proliferative action of estrogen on lactotrophs [17], and increased tumoral VEGF expression was observed during estrogen-induced prolactinoma development in rats [18]. These data indicate that even though the role of angiogenesis in pituitary adenomas is contentious, VEGF might contribute to adequate temporal vascular supply.

VEGF expression has been described in all cell types in the normal pituitary, with greater expression in somatotrope and follicle-stellate cells. Using immunohistochemistry, higher VEGF expression has been shown in the normal human pituitary gland compared with adenomas [19], while the opposite has also been published [20]. In a group of ACTH and GH secreting adenomas, pituitary carcinomas had the strongest VEGF immunoreactivity, and furthermore, VEGF expression was related to suprasellar extension [19]. It was also described that VEGF and KDR expression were expressed more on tumors with extrasellar growth than intrasellar ones, suggesting they could be markers for poor outcome after partial tumor resection [21]. On the other hand, Viacava et al. [20] found no differences in VEGF expression among tumors of different histotypes, and McCabe et al. comparing VEGF in a series of adenomas composed of 77% nonfunctioning adenomas and only 4% of prolactinomas found the highest expression in nonfunctioning adenomas and GH producing adenomas [22]. Elevated serum VEGF concentrations have been demonstrated in patients harboring pituitary tumors, and approximately 90% of human pituitary tumors cultured* in vitro* show measurable VEGF secretion.

In a cohort of pituitary adenomas we found that VEGF protein expression was higher in dopamine agonist resistant prolactinomas compared to nonfunctioning GH and ACTH secreting adenomas [23]. This finding may be related to the high percentage of macroprolactinomas in the series studied, as it has been described that macroprolactinomas are significantly more vascularized than microprolactinomas. Furthermore, lower VEGF found in ACTH-producing adenomas may be consistent with the finding that VEGF production can be suppressed by glucocorticoids which are potent inhibitors of VEGF production* in vitro* [24].

Most studies reveal that sex, age, or even rate of recurrence does not influence VEGF expression in pituitary tumors.

Cyclin-dependent kinase 5 (CDK5) regulates the activities of various proteins and cellular processes in the nervous system and is present in normal human pituitary and in pituitary tumors. It has been shown that increased CDK5-mediated VEGF expression might play a crucial role in the development of pituitary adenomas. These results suggest that roscovitine and other CDK5 inhibitors could be useful as antiangiogenic drugs in invasive pituitary adenomas [25].

These data indicate angiogenesis in the pituitary tumors, as well as in other endocrine neoplasms, probably reflects the basic observation that tumors require neovascularization to grow; however, the changes that occur may be somewhat different from some other tissues that are less vascularized in the nonneoplastic state. Some data suggest that VEGF may prolong cell survival by inducing expression of the antiapoptotic protein bcl-2 in pituitary adenomas, suggesting that part of its angiogenic activity is related to protection of endothelial cells from apoptosis. VEGF has also been associated with intratumoral hemorrhage [26] and might also participate in the occurrence of pituitary peliosis, a form of vasculogenic mimicry. Peliosis may be linked to the permeabilizing function of this growth factor, and to the increased fenestration induced in blood vessels stimulated by VEGF overexpression. Peliosis occurrence has been related to high VEGF expression in hepatocarcinogenesis, spleen damage, and in a lethal hepatic syndrome in mice. This process may be seen in prolactinomas and other pituitary adenomas, though it usually goes unrecognized.

### 2.3. Fibroblast Growth Factor-2

Another potent angiogenic factor is basic fibroblast growth factor-2 (basic FGF, or FGF2). It was originally isolated from the bovine pituitary and has a pleiotropic activity affecting both vasculature and parenchyma cell proliferation and differentiation [27]. It belongs to a large family of heparin-binding growth factors comprising at least 22 structurally related members. FGF2 expression is complex; at least four FGF2 isoforms (18, 22, 22.5, and 24 kDa) in human and three (18, 21, and 22 kDa) in mouse are synthesized through alternative translation initiation from CUG codons. The 18 kDa isoform is predominantly cytoplasmic but can also be found in the extracellular matrix, while the higher-molecular-weight isoforms are localized in nuclei and ribosomes. The 18 kDa FGF2 isoform is highly expressed in the normal human pituitary, while pituitary adenomas produce predominantly the 24 kDa form [28]. More recently, a 34 kDa isoform was reported, with the most upstream CUG codon among all FGF2 forms [29]. None of the isoforms have a typical secretory signal sequence, but alternative pathways have been described for their export from the cell.

The biological effects of FGF2 are mediated through four high-affinity transmembrane receptors (FGFR1–FGFR4) that have intrinsic tyrosine kinase activity [30]. They can be found on a wide variety of cell membrane surfaces including endothelial cells where FGF2 exerts its proangiogenic functions.

### 2.4. Fibroblast Growth Factor-2 and FGFR1 in the Pituitary

FGF2 participates in pituitary development and proliferation and regulates hormone synthesis and secretion, affecting prolactin and TSH production. It is mainly produced by folliculostellate cells [31], although somatotropes and gonadotropes have also been reported to be sources of this growth factor.

FGF2 participates in estradiol-mediated prolactinoma induction in rats under both physiological and pharmacological conditions [32–34]. FGF2 is also expressed by human pituitary adenoma cells* in vitro*, and high levels of serum FGF2 were found in patients bearing pituitary tumors, declining following surgical adenomectomy [35].

In the case of a giant invasive prolactinoma with loss of response to dopamine agonist therapy we have reported strong immunoreactivity for both angiogenic factors VEGF and FGF2, as well as immunoreactivity for the endothelial cell marker CD31 indicating high vascularization of the adenoma [36].

FGFR1 is found in the normal human pituitary as well as in pituitary adenomas, and its mRNA was described in the rat neural and anterior lobe. Furthermore, FGFR1 has been proposed as a candidate marker of pituitary tumors together with FGF2 and pituitary tumor transforming gene (PTTG); indeed, the FGF2 receptor FGFR1 was found to be highly expressed in pituitary tumors compared to the normal gland [37]. Furthermore, significantly increased* Fgfr1* mRNA expression was described in functioning tumors that invaded the sphenoid bone compared with those that did not, thus raising the possibility of using the FGFR1 as a molecular marker of tumor biological behavior [37]. On the other hand, it has also been determined that cytoplasmic FGFR1 immunoreactivity was inversely correlated with maximum pituitary tumor diameter [38].

Some proteins and genes related to FGF-2 have been linked to the development of prolactinomas such as FGF-4, PTTG, thrombospondin, FGF2 antisense RNA, or truncated FGFR4.

*FGF4*. DNA derived from human prolactinomas codifies for transforming activity in heterologous cells and has sequences in close resemblance with those of* hst* gene. Overexpression of* hst* gene leads to increased production of FGF4. Shimon et al. [39] demonstrated the function of the* hst* gene in rat lactotrope tumor formation and prolactin secretion. They were able to show that lactotropes in 5 of 14 prolactinomas stained strongly with anti-FGF-4 monoclonal antibodies, and the immunoreactive hst product in adenoma cells was observed in invasive prolactinomas [40]. These findings imply a role of* hst* gene, and its product FGF4, in cellular proliferation, growth, and aggressive behavior in prolactinomas.

*PTTG*. Pituitary tumor transforming gene (*Pttg*), located on chromosome 5q33, has been shown to be tumorigenic* in vivo*, by regulating FGF2 secretion and inhibiting chromatid separation [41, 42]. Estrogen promotes experimental prolactinoma development via induction of a pituitary tumour transforming gene (*pttg*) [43]. Patients with prolactinomas which were responsive or unresponsive to dopamine agonists had similar pituitary* PTTG* mRNA levels [43].

Recently a meta-analysis suggested that PTTG expression may be associated with tumor invasiveness and microvessel density of pituitary adenomas [44].

*Thrombospondin-1*. TSP-1 is a modular glycoprotein secreted by different cell types, including endothelial cells. It is composed of multiple active domains that bind to soluble factors, cell receptors, and extracellular components. TSP-1 was the first endogenous inhibitor of angiogenesis to be identified and its effect is due, at least in part, to its capacity to bind FGF2 [45]. TSP-1 is reduced in estrogen induced pituitary adenomas [46] and TSP-1 agonists can inhibit experimental prolactinoma development and angiogenesis in rats [46, 47].

*FGF2 Endogenous Antisense (GFG) RNA*. In* Xenopus laevis* oocytes, a 1.5 kb* FGF2* antisense (GFG) RNA complementary to the third exon and 3′UTR of FGF-2 mRNA has been implicated in FGF2 mRNA regulation. The human homolog has been localized to the same chromosomal site as FGF2 (chromosome 4, JO4513 adjacent to D4S430), confirming this as a human endogenous antisense gene. This GFG antisense gene also encodes a 35 kDa protein and regulates cell proliferation and hormone secretion. Pituitary tumors have been shown to express FGF2; GFG protein levels are higher in the normal gland than in most tumors, and aggressive pituitary adenomas appear to express more FGF-2 than GFG mRNA [48].

*Truncated FGFR4*. Altered FGF receptor expression has been found in pituitary adenomas [48], and FGFR4 undergoes alternative transcription initiation in pituitary adenomas, giving rise to an oncogenic protein in pituitary adenomas of various subtypes. Expression of this pituitary tumor-derived- (ptd-) FGFR4 protein is more frequent in macroadenomas than in microadenomas and correlates with the Ki-67 labeling index. Some data suggest that ptd-FGFR4 alters cell adhesion by a mechanism that explains the loss of reticulin, which is the hallmark of pituitary adenomas.

Taken together, these data suggest that deregulated FGF/FGFR system function plays a role in pituitary tumorigenesis and particularly in prolactinoma development.

### 2.5. Markers of Vascular Development in Pituitary Tumors: CD31, CD34, Endocan, and Nestin

Different markers of microvascular density (MVD) such as CD31 and CD34, Factor VIII (factor eight-related antigen), and Ulex europaeus agglutinin I, and nestin have been used to evaluate angiogenesis.

CD31 and CD34, both endothelial cell antigens, are sensitive markers of microvessels. They stain the majority of tumor vessels, both mature and new vessels. Even though antibodies to CD31 are not completely specific for endothelial cells, as they may also detect plasma cells, they are widely used for MVD appraisal, and results generally correlate with those obtained with CD34. Using these endothelial cell markers, some authors have found more prominent vasculature in prolactinomas, and others found that these tumors had the lowest while TSH secreting adenomas had the highest MVD [49]. It has also been reported that ACTH secreting tumors had the lowest MVD [4, 50], while other authors found that GH secreting adenomas had the lowest [3, 51, 52] or the highest MVD [4]. Finally, some authors did not find any significant difference in MVD between the hormonal subtypes [20, 53]. These results point to the complexity of evaluation of vascularity in the adenomatous pituitary.

Interestingly, we described a high correlation of VEGF and CD31 expression for prolactinomas and nonfunctioning adenomas [23]. The strong positive association of VEGF and CD31 expression found in human pituitary adenomas suggests the participation of tumor vascularization in adenoma development. Even so, this is in contrast to results published by other authors in which MVD did not correlate with VEGF expression.

Furthermore, Endocan, a new marker of vascular endothelial cells from cancers and closely related to tumor angiogenesis, is exclusively expressed in CD34-positive vascular endothelial cells in pituitary adenomas and significantly elevated in macroadenomas compared with microadenomas [54].

On the other hand, proliferation markers (PCNA and Ki67) do not correlate with the angiogenic markers CD31 and VEGF, as described by us and others [5, 49, 52, 55–57]. This suggests that the rate of epithelial and tumor cell proliferation in pituitary tumors is not directly related to neovascularization, and other factors, such as primary genetic alterations or alteration of apoptotic pathways, may directly affect the rate, invasiveness, and tumor behavior. To this respect, a positive relationship was observed, between the expression of bcl-2, an antiapoptotic protein, and increasing MVD suggests an association between angiogenesis and cell survival [56, 58].

In a recent work we found that adenomas had a lower vascular area compared to normal pituitary tissue, but, interestingly, pituitary adenomas had significantly more small vessels than control pituitaries [59].

Low vascularization is a peculiar situation for tumors despite their benign nature, as even premalignant lesions like precarcinomas of the cervix and breast have increased MVD [60, 61]. However, some benign tumors that hardly ever progress to malignancy were reported with lower vascular density when compared to normal tissue [62–64]. Nevertheless, even though vascular area was lower in pituitary tumors compared to the normal gland, vessel size proportion was markedly different in the normal and tumoral pituitary. This suggests that the increased percentage of small vessels in adenomas may be the predominant feature associated with angiogenesis. In accordance with our results, Itoh et al. suggested that angiogenesis in the tumoral pituitary may occur with changes in diameter and shape of blood vessels [50].

With regard to the relation between MVD and sex or age of the patients, contradictory findings have also been reported. Jugenburg et al. [3] reported no significant correlations, whereas Turner et al. [6] found tumor MVD clearly decreased with age in GH producing adenomas, and there was a trend in other tumor types from older patients to have lower MVD. In contrast, a positive correlation between age and MVD has also been reported. We described that, in pituitary adenomas, CD31 expression was not different between sexes and did not correlate with patients' age when all adenomas were considered. Nevertheless, if only nonfunctioning adenomas were analyzed, we found a positive correlation of CD31 with increasing age [23], in agreement with other authors [52], and therefore age may have an influence on the extent of neovascularization of nonfunctioning adenomas.

An additional reliable marker of neovascularization is nestin. It is a class VI intermediate filament protein that participates in cytoskeleton formation and has been found in endothelial cells of newly formed blood vessels of developing organs [65]. It was originally described as a neuronal stem/progenitor cell marker in cells of the developing central nervous system [66]. In particular, it has been reported that nestin-containing cells in the pituitary gland play an important role in its cellular and morphological plasticity throughout life [67]. Moreover, nestin expression was detected in endothelial cells of pituitary adenomas and in a carcinoma sample [68].

We found that nestin expression was evidenced only in the adenomatous pituitaries and correlated positively with the percentage of small vessels and negatively with years since the first diagnosis of pituitary adenoma [59]. Nestin has been detected in various neoplasms such as astrocytomas and malignant gliomas, including glioblastoma multiforme [69] and prostate cancer [70]. In these tumors it was generally expressed in immature endothelial cells generated in the course of angiogenesis [65, 71] and in the adult human pancreas nestin localized in endothelial cells predominantly of small caliber [72]. In our cohort of adenoma samples nestin localized mainly associated with blood vessels, and the inverse correlation of nestin with years of tumor evolution or large blood vessels may suggest that nestin is expressed mainly in the setting of angiogenesis, and not in the quiescent endothelium, as previously suggested for other neoplasms [73]. Small vessels probably represent the newly formed blood vessels during pituitary adenoma generation. Indeed, nestin expression was evidenced only in newly formed capillaries growing into the infarcts and not in the necrotic capillaries, during pituitary infarction or apoplexy [74].

Therefore, this stem cell marker may be associated with endothelial cell development in pituitary adenomas.

### 2.6. Dopamine D2 Receptors

A relationship between D2Rs and endothelial cell proliferation within tumors has been proposed. Dopamine and other related catecholamine neurotransmitters that interact with the D2R selectively inhibit VEGF-induced angiogenesis and inhibit the growth of malignant tumors as well as the vascular permeabilizing and angiogenic activities of VEGF [75]. Besides, in two outbred lines of Wistar rats, which present high and low dopaminergic reactivity, respectively, VEGF expression was lower in the first group, and this group was more resistant to tumor implantation and developed significantly fewer lung metastases [76].

These data, as well results obtained in animal models, indicate that the D2R is linked to pituitary VEGF expression. In dopamine agonist resistant prolactinomas a decrease in number or function of D2Rs has been proposed [77], and we have found highly expressed VEGF in a dopamine agonist resistant giant prolactinoma [36], as well as in a cohort of dopamine agonist resistant macroprolactinomas [23].

## 3. Mutant Animal Models of Dopamine Agonist Resistant Prolactinomas

Prolactin secreting adenomas are the most frequent type among pituitary tumors. Patients usually present endocrinological symptoms resulting from hyperprolactinemia and, less commonly, in the case of macroprolactinomas, they have visual defects due to compression of the optic chiasm. Macroprolactinomas are benign, slowly proliferating tumors, although they may be locally highly aggressive, particularly in males, and invade adjacent structures. Giant prolactinomas (tumor volume exceeding 4 cm in diameter and/or with prolactin levels higher than 3000 ng/mL and mass effect), a rare subcategory of macroprolactinomas, remain one of the greatest challenges in neurosurgery. Because of invasive growth, giant adenomas can compress or destroy adjacent structures, resulting in neurological dysfunction and cavernous sinus compression. D2Rs are found in pituitary lactotropes, where they mediate the tonic inhibitory control that dopamine exerts on prolactin synthesis and release, and therefore pharmacological therapy with dopamine agonists remains the mainstay of treatment. This therapy is effective in more than 85% of patients with prolactin-secreting pituitary tumors. A minority of patients show no primary response to either bromocriptine or cabergoline [78], and the development of dopamine agonist resistance in an initially responsive prolactinoma is unusual. A decrease in number or function of D2Rs has been proposed in dopamine agonist resistance [79, 80]. In these cases, tumors tend to be invasive and aggressive and may require extirpation [78]; therefore, an alternative target would be desired.

The physiological significance of dopamine inhibitory control in lactotrope proliferation and secretory activity has been appreciated in mice lacking D2Rs generated by targeted mutagenesis (*Drd2* ^−/−^) [9]. Female* Drd2* ^−/−^ mice have pituitary hyperplasia, chronic hyperprolactinemia, and provide an experimental model for dopamine agonist resistant prolactinomas [81]. In* Drd2* ^−/−^ mice highly vascularized adenomas develop after 16 months of age, especially in females, but also in males [82].

Analysis of* Drd2* ^−/−^ mice also revealed the unexpected importance of D2Rs in the regulation of the growth hormone (GH) axis and control of body size, a differential phenotype that is considerable in males.* Drd2* ^−/−^ mice display a shortfall of pituitary somatotropes and reduced GH and IGF-I serum levels and are dwarfs [83, 84]. Somatotrope shortfall and dwarfism of* Drd2* ^−/−^ mice are related to the lack of central D2Rs which regulate growth hormone-releasing hormone or somatostatin function [85]. Therefore, by conducting a functional dissection strategy based on cell-specific* Drd2* inactivation in lactotropes we developed a strain of transgenic mice expressing* cre* from a mouse prolactin gene promoter, Tg(Prl-cre)^1Mrub^ to eliminate D2Rs from pituitary lactotropes (LacDrd2KO). LacDrd2KO female mice exhibit chronic hyperprolactinemia, marked pituitary hyperplasia, and a preserved GH axis and therefore provide a cleaner mutant model to study the generation and regulation of dopamine agonist resistant prolactinomas without the confounding effect of central D2Rs.

## 4. Angiogenic Factors in Mouse Models of Dopamine Agonist Resistant Prolactinomas

### 4.1. VEGF

D2R knockout (*Drd2* ^−/−^) mice generated by targeted mutagenesis and lacDrd2KO mice, generated by Cre LoxP technology, have chronic hyperprolactinemia, pituitary hyperplasia, and provide experimental models for dopamine agonist resistant prolactinomas [10, 81]. In* Drd2* ^−/−^ mice highly vascularized adenomas develop after 16 months of age, especially in females, but also in males [82]. Prominent vascular channels, as well as extravasated red blood cells not contained in capillaries or peliosis, are common findings in the hyperplastic and adenomatous* Drd2* ^−/−^ pituitaries. Peliosis has been found in different tumors that secrete VEGF. In accordance, VEGF mRNA and protein expression are increased in pituitaries from* Drd2* ^−/−^ female mice [86]. In lacDrd2KO female mice hyperplastic pituitaries also showed enhanced vascularization and VEGF content, with no shortfall of somatotropes, as in the global knockout model [10]. These results support the notion that defective function of lactotrope D2Rs increases VEGF expression and may participate in pituitary angiogenesis of prolactinomas.

Pituitary VEGF production is stimulated by estrogen in rat pituitaries and the somatolactotrope cell line GH3. Nevertheless, estrogen levels are not increased in* Drd2* ^−/−^ or lacDrd2KO female mice, indicating that increased pituitary VEGF expression is mainly dependent on the lack of dopaminergic control. In experiments with wild-type female mice we found that prolonged treatment with the D2R antagonist, haloperidol, enhanced pituitary VEGF protein content and prolactin release [86], and there was a significant correlation between pituitary VEGF levels and serum prolactin after haloperidol treatment. These results support the notion that dopamine acting at the D2R inhibits pituitary VEGF expression.

**Figure 1:**
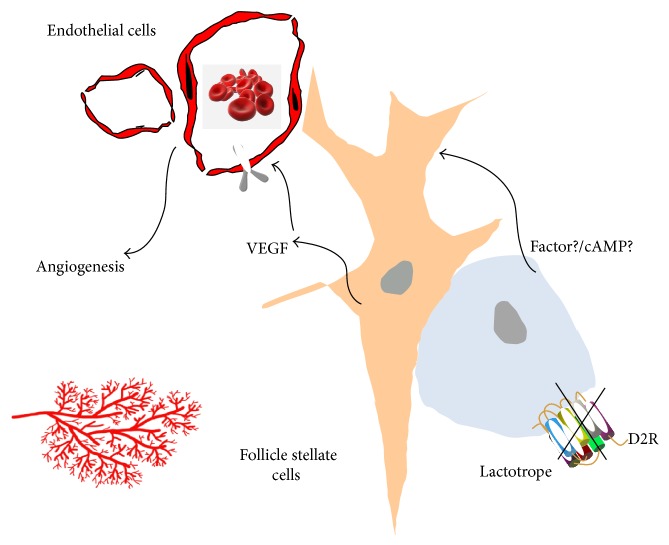
Disruption of the D2R from lactotropes induces paracrine VEGF secretion from follicle stellate cells. VEGF acts on VEGFR2 receptors on endothelial cells to induce pituitary angiogenesis.

Interestingly, we found that the main source of VEGF in the hyperplastic pituitary was follicle stellate cells and not lactotropes [86]. Follicle stellate cells represent 5 to 10% of pituitary cells and are an important component of paracrine communication within the pituitary. They are detected by their content of the glial protein S100, and they form follicles, are star shaped, and have long processes in between the secretory cells of the pituitary. They also contain FGF-2, follistatin, and interleukin 6. Because D2Rs have been described in lactotropes and not in follicle stellate cells, it may be inferred that a paracrine-derived factor from lactotropes is acting on follicle stellate cells to increase VEGF expression (Figure 1).

### 4.2. FGF2

In the hyperplastic pituitaries of* Drd2* ^−/−^ mice, FGF-2 promoted prolactin secretion and cellular proliferation, and, interestingly had a differential subcellular distribution compared to that of wild-type pituitaries, which could be associated with different biological roles of this angiogenic factor in both genotypes [87]. Nevertheless, pituitary FGF2 content was not increased in this model.

### 4.3. PTTG

When compared to female wild-type mice, pituitaries from female* Drd2* ^−/−^ mice had decreased PTTG concentration [43]. PTTG did not correlate with prolactin levels or tumor size in animal models of prolactinoma, and its pituitary content was not related to a decrease in dopaminergic control of the lactotrope, but it was positively influenced by estrogen action at the pituitary level [43].

**Figure 2:**
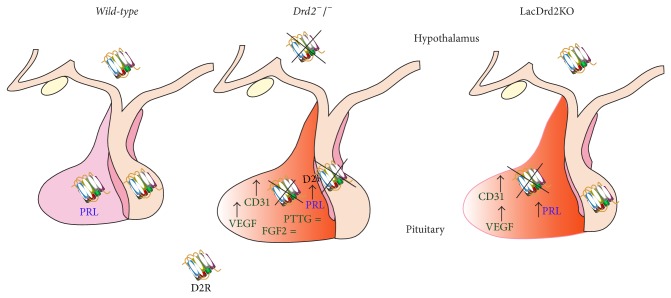
Total (*Drd2* ^−/−^) or lactotrope (LacDrd2KO) disruption of D2R evokes and increases in pituitary VEGF and CD31, with no alteration of PTTG or FGF2 content. Pituitary angiogenesis correlates with pituitary hyperplasia and increased prolactin synthesis and release.

Taken together these results suggest that angiogenesis of pituitary tumors in the* Drd2* ^−/−^ mice does not depend on FGF-2 and PTTG expression. Instead, the development of new blood vessels seems to be dependent on VEGF, which is increased due to the absence of dopaminergic control (Figure 2). The pattern of expression of the angiogenic factors would determine the angiogenic phenotype of the prolactinomas and probably reflect the benign nature and slow growth rate of these tumors, compared to highly aggressive tumors such as melanoma in which most of the angiogenic factors are upregulated.

## 5. Antiangiogenesis in Pituitary Tumors

VEGF and its receptor may become supplemental therapeutic tools in dopamine-resistant prolactinomas. In this regard, in recent years, antiangiogenesis has been publicized as a novel alternative or supplement to conventional cancer therapy, and a variety of regimens that prevent tumor angiogenesis and/or that attack tumor blood vessels have met with remarkable success in treating mouse cancers [88]. There has been a great interest in the targeting of tumor vasculature and the development of antiangiogenic agents, which interrupt tumor's supply of oxygen and nutrients. Treatment with anti-VEGF antibodies significantly inhibited growth of several tumor cells lines and has been approved by the FDA for a combinatorial treatment with chemotherapy for metastatic colorectal cancer, nonsmall-cell lung cancer, metastatic breast cancer, and more recently glioblastoma multiforme and renal cell carcinoma [89]. However not all trials have been positive [90, 91], indicating that individual characteristics of different tumors should be studied. Furthermore, resistance to anti-VEGF therapies develops after some months of treatment in most patients. Therefore, despite the spectacular successes reported in the treatment of mouse tumors, the first clinical trials were discouragingly negative. This could be related to the fact that most of the patients treated in the beginning had advanced disease and had already failed conventional treatments. Also, antiangiogenesis therapy differs fundamentally from chemotherapy, and optimal implementation is still needed.

In* Drd2* ^−/−^ female mice using two strategies with anti-VEGF compounds we demonstrated that VEGF is required for the maximal growth of this mouse model of dopamine agonist resistant prolactinomas [7]. Local therapy with VEGF-TRAP or a systemic treatment with a monoclonal antibody targeting murine VEGF resulted in substantial tumor and prolactin inhibition in hyperplastic pituitaries from* Drd2* ^−/−^ female mice. Additionally, there were significant decreases in vascularization and proliferation index induced by both anti-VEGF strategies in the pituitary tumors. These data suggest that the antiangiogenic treatments were effective in inhibiting the growth of primary dopamine resistant prolactinomas as well as the transplanted adenomas.

Furthermore, in an aggressive prolactinoma generated in the multiple endocrine neoplasia 1 mouse model, Mab G6-31, a monoclonal anti-VEGF antibody, inhibited the growth of the intracerebrally injected pituitary adenoma and reduced prolactin levels [92].

In lactotrope hyperplasia induced by a synthetic estrogen, treatment with ABT-510 and ABT-898, two thrombospondin analogs with antiangiogenic properties, counteracted pituitary size and serum prolactin increase, and decreased tumor vasculature [47].

Curcumin (diferuloylmethane), a polyphenolic compound derived from the spice plant* Curcuma longa*, displays multiple actions on solid tumours including antiangiogenic effects. Curcumin dose-dependently inhibited basal VEGF secretion in corticotrope AtT20 mouse and lactosomatotrope GH3 rat pituitary tumour cells as well as in human pituitary adenoma cell cultures [93] indicating its potential as an antiangiogenic agent in pituitary adenomas.

In humans, antiangiogenic therapy was used in the treatment of an aggressive pituitary tumor. An aggressive silent corticotrope cell pituitary adenoma, subtype 2, that progressed to carcinoma despite temozolomide administration was treated with the anti-VEGF monoclonal antibody bevacizumab for 26 months with stabilization of disease as documented on serial MRI and PET scans [94].

The present findings in murine animal models and humans suggest that antiangiogenic therapy may represent a complementary option in the treatment of aggressive pituitary tumors.

## 6. Conclusions

In pituitary adenomas an altered expression of angiogenic growth factors and their receptors has been observed [48, 57, 95–97]. Although it is unlikely that these alterations play a causative role in pituitary tumor pathogenesis, intratumoral changes of these factors and their receptors may result in a permissive microenvironment that contributes to excessive hormone production and loss of growth control in pituitary adenomas.

Each pituitary tumor of clonal origin represents the multifactorial result of failure of different regulatory events. In this regard, pro- and antiangiogenic growth factors, such as FGF-2, VEGF, and others, may determine the final angiogenic phenotype of pituitary tumors and thus subsequent tumor behavior. Furthermore, the study of angiogenic factor expression in aggressive prolactinomas with resistance to dopamine agonists will yield important data in the search of therapeutical alternatives.

We conclude that angiogenesis is an active process in these tumors, in spite of their low total vascular area when compared to nontumoral pituitary. Understanding the role of angiogenesis in their development may facilitate therapeutical management in the cases of adenomas that cannot be controlled by conventional therapy.
