# Corrigendum to “Angiogenesis in Pituitary Adenomas: Human Studies and New Mutant Mouse Models”

**DOI:** 10.1155/2020/8978014

**Published:** 2020-07-18

**Authors:** Carolina Cristina, Guillermina María Luque, Gianina Demarchi, Felicitas Lopez Vicchi, Lautaro Zubeldia-Brenner, Maria Ines Perez Millan, Sofia Perrone, Ana Maria Ornstein, Isabel M. Lacau-Mengido, Silvia Inés Berner, Damasia Becu-Villalobos

**Affiliations:** ^1^Instituto de Biología y Medicina Experimental, CONICET, Vuelta de Obligado 2490, 1428 Buenos Aires, Argentina; ^2^CITNOBA (CONICET-UNNOBA), Universidad Nacional del Noroeste de la Provincia de Buenos Aires, Monteagudo 2772, Pergamino, 2700 Buenos Aires, Argentina; ^3^Servicio de Neurocirugía, Clínica Santa Isabel, Avenida Directorio 2037, C1406GZJ Buenos Aires, Argentina; ^4^Servicio de Neurocirugía, Hospital Santa Lucía, Avenida San Juan 2021, C1232AAC Buenos Aires, Argentina

The article titled “Angiogenesis in Pituitary Adenomas: Human Studies and New Mutant Mouse Models” [1] reused wording from a book chapter published the same year by the same author group, “Prolactinomas: Role of VEGF, FGF-2 and CD31” in Tumors of the Central Nervous System [2], which was not cited. The review brought together the results of the authors' previous experimental work on angiogenesis and pituitary adenomas in the context of the literature.
